# Supplementary figures and images for: Development of in-house, indirect ELISAs for the detection of SARS-CoV-2 spike protein-associated serology in COVID-19 patients in Panama
Source: PLoS One. 2021 Sep 14;16(9):e0257351. doi: 10.1371/journal.pone.0257351 (PMC8439474; doi:10.1371/journal.pone.0257351)

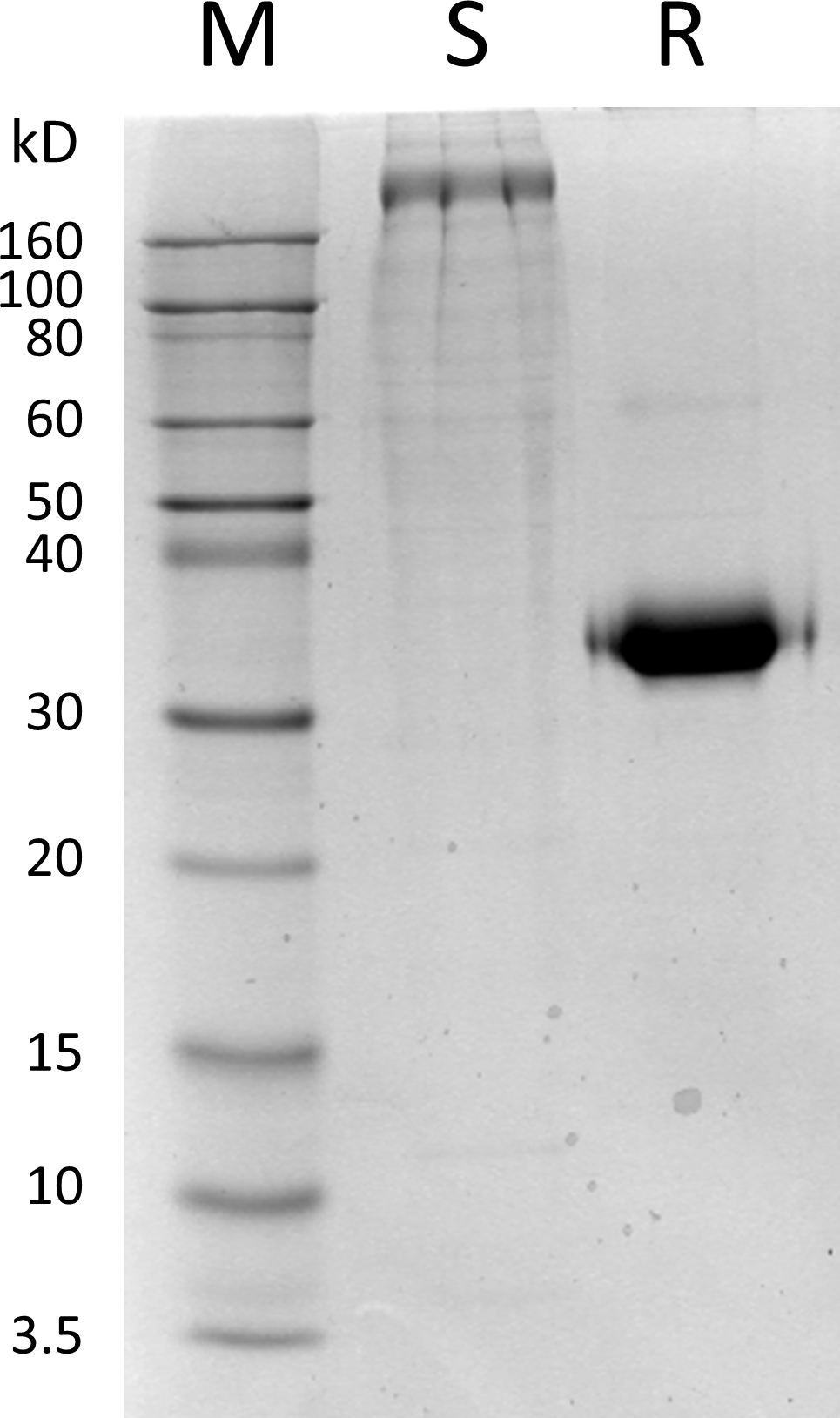

Supplement: S1 Fig — M, Protein size standards in kilodalton (kD); S, recombinant SARS-CoV-2 spike protein; R, recombinant SARS-CoV-2 spike fragment, corresponding to the Receptor Binding Domain (RBD). (TIF) [file pone.0257351.s001.tif]

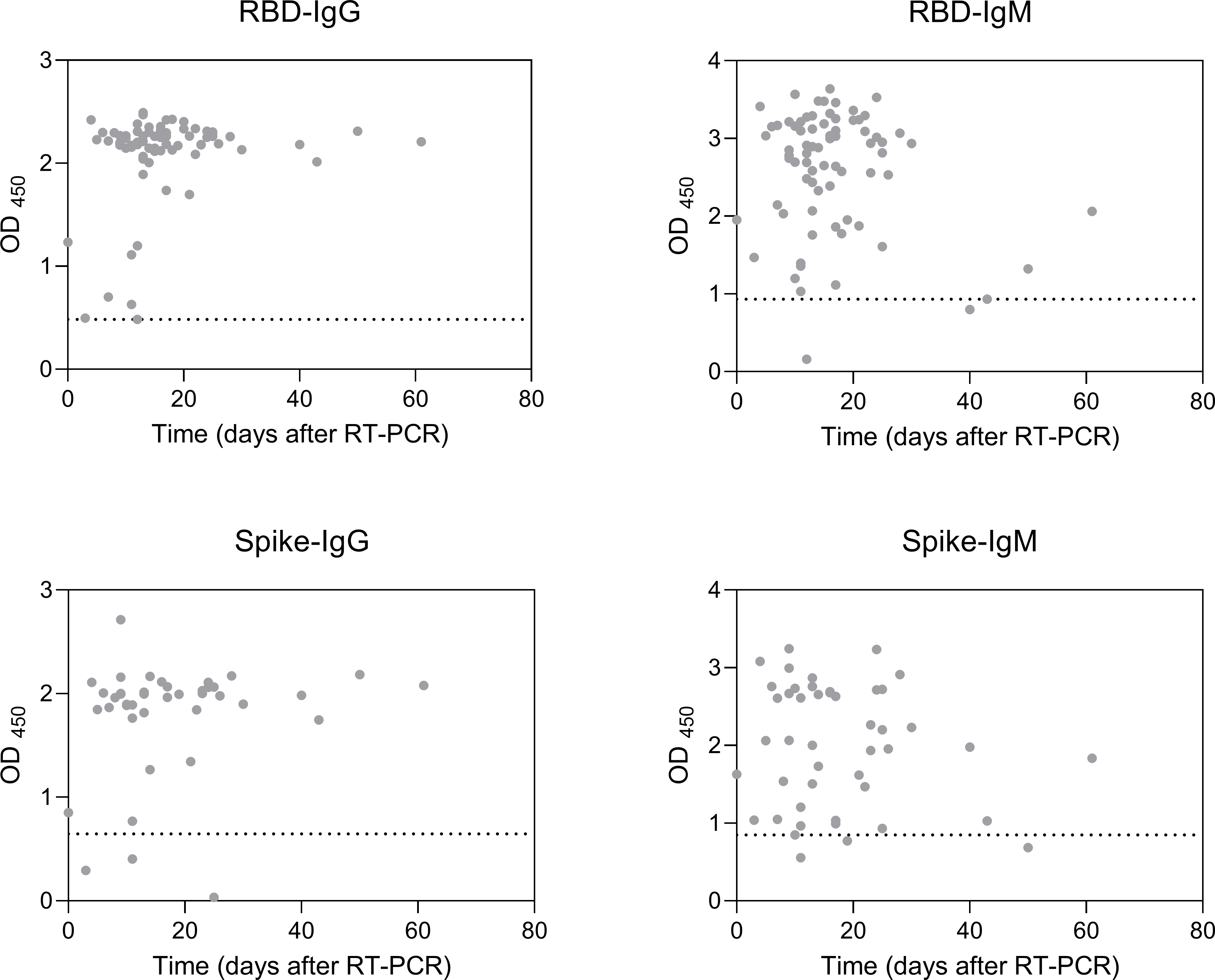

Supplement: S2 Fig — RBD: recombinant spike receptor binding domain. Spike: recombinant full Spike protein. Dotted lines represent the cutoff value for each ELISA. (TIF) [file pone.0257351.s002.tif]
